# Supplementary material for: Characterization of neonatal opioid withdrawal syndrome in Arizona from 2010-2017
Source: PLoS One. 2021 Jun 3;16(6):e0248476. doi: 10.1371/journal.pone.0248476 (PMC8174702; doi:10.1371/journal.pone.0248476)

**Table S1. Summary of non-geographic data used in this study by figure.** The Mothers^O^ column contains data for all mothers who were dependent on opioids at the time of giving birth. The Infants^NAS^ column contains data for all infants who had NAS. Totals at the end of each category are not the same for all categories due to missing data or because some patients reported atypical categories (e.g. payer was workers compensation or individual was a foreign national).


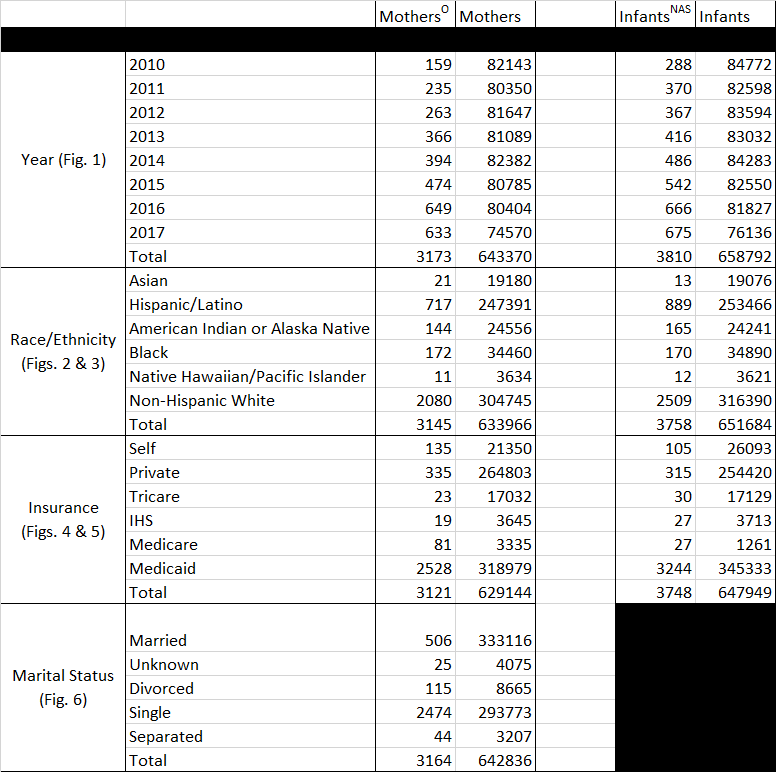

Supplement: S1 Table — The MothersO column contains data for all mothers who were dependent on opioids at the time of giving birth. The InfantsNOWS column contains data for all infants who had NOWS. Totals at the end of each category are not the same for all categories due to missing data or because some patients reported atypical categories (e.g. payer was workers compensation or individual was a foreign national). (DOCX) [file pone.0248476.s001.docx]
