# Supplementary material for: Characterization of neonatal opioid withdrawal syndrome in Arizona from 2010-2017
Source: PLoS One. 2021 Jun 3;16(6):e0248476. doi: 10.1371/journal.pone.0248476 (PMC8174702; doi:10.1371/journal.pone.0248476)
Supplement: Attachment — Submitted filename: PLOS One NAS Aug21 2020.docx [file pone.0248476.s002.docx]

RE: Review of PONE-D-20-17306; Characterization of Neonatal Abstinence Syndrome in Arizona from 2010-2017

DATE: August 21, 2020

Thank you for the opportunity to review this manuscript, which summarizes the occurrence of NAS over 8 years in Arizona. The data are unique in that the they span before and after the installation of a NAS surveillance system. The data also end in 2017, the year that the AZ governor declared that opioid use disorder had reached epidemic levels. I believe there is great potential for this paper, but have recommendations that may enhance its quality. I will review these in the order that they appear.

ABSTRACT

The structure of the abstract should be revisited so that the lit review is completed prior to stating the purpose of the study. Following the research justification and purpose, the data description and results would follow. Currently, after the research purpose, the authors revisit literature justification prior to moving into the results. This is an awkward sequencing.

INTRODUCTION

Line 56: Treatment protocol for NAS does not seem relevant for this paper unless you wish to link the procedure to length of stay. Clarify the importance of treatment in relation to the study or delete.

Line 69-76: It is unclear how the discussion of prevention, screening for NAS, and standardization of care for infants with NAS is relevant to this manuscript. These issues may be worked into the discussion perhaps but they do not assist in justifying the importance of the study. I recommend the deletion of this paragraph. More attention could be given to what we know about the demographics of mothers with infants with NAS, and their comorbidities, nationally or in other states.

Line 75: Note, the *early* identification of NAS cases can be challenging as some infants do not exhibit signs of withdrawal until days after their birth.

Line 93: Clarify why the initiation of the surveillance system would lead to a hypothesized increase in NAS cases. Given that NAS is determined based on the medical codes, the codes should remain consistent for billing purposes – both before and after the surveillance system. Based on existing data, it seems this increase was typical, until greater efforts to intervene were implemented.

METHODS

Line 142: The study includes only non-tribal PCAs. In attempts to better understand the state of AZ, the exclusion of the tribal areas diminishes the ability to address the study purpose. The possible impact of not including tribal areas should be explored further in the Discussion, based on what is understood about NAS in the tribal communities.

RESULTS and DISCUSSION

Line 164: The cases of NAS are not clearly laid out to test whether there was an increase in cases after the surveillance system was implemented. As a proposed hypothesis this should be explicitly tested. (e.g., in relation to Figure 1).

Line 191: The race, ethnicity, marriage status, and insurance coverage are very comparable to the data across the US. Ideally such data from earlier studies would be reflected in the Introduction.

Length of stay can vary greatly in whether infants are released earlier to complete weaning at home, use of rescue dosing, and the inclusion or exclusion of complementary treatment approaches. The variability of the AZ treatment approaches should be discussed in the Discussion.

Line 262: This is an example of the Discussion lurking into the Results. This is not in keeping with manuscript sections. Please keep the Results in the first section and the Discussion points would then follow.

Line 296: Given the variability of the comorbid conditions, it seems that this may contribute to challenges related to standardization of care. This needs to be revisited and better clarified.

In sum, I believe these data have much to contribute to our understanding of NAS in relation to both infants and their mothers. However, opportunities to strengthen this contribution remain. Toward this end, I hope these comments are beneficial
